# Evaluation of *Leea rubra* Leaf Extract for Oxidative Damage Protection and Antitumor and Antimicrobial Potential

**DOI:** 10.1155/2021/7239291

**Published:** 2021-10-06

**Authors:** Nibedita Das, Sanowar Hossain, Jaytirmoy Barmon, Shahnaj Parvin, Mahadi Hasan, Masuma Akter, Ekramul Islam

**Affiliations:** ^1^Rajshahi Medical College, Rajshahi 6100, Bangladesh; ^2^Department of Pharmacy, Pabna University of Science and Technology, Pabna 6600, Bangladesh; ^3^Department of Pharmacy, Varendra University, Kazla, Rajshahi, Bangladesh; ^4^Department of Pharmacy, University of Rajshahi, Rajshahi 6205, Bangladesh

## Abstract

**Background:**

The leaves of *Leea rubra* contain an abundance of phenolic constituents and have medicinal uses as antipyretic and diaphoretic agents and are also used in the treatment of stomach ache, rheumatism, arthritis etc. In spite of the traditional uses, data on the scientific evaluation of the plant are not sufficient. So, the present study was designed to evaluate the protective role of the extract against oxidative damage to DNA and human erythrocytes as well as antitumor and antibacterial activities against some resistant bacteria.

**Methods:**

The protective activity of the ethyl acetate fraction (EAF) of the extract was investigated by evaluating the inhibition of oxidative damage of pUC19 plasmid DNA as well as hemolysis and lipid peroxidation damage to human erythrocytes induced by 2,2′-azobis-2-amidinopropane (AAPH). Antitumor activity was assessed by evaluating the percentage inhibition of cell growth, morphological changes of Ehrlich's ascites carcinoma (EAC) cells, and hematological parameters. Antimicrobial activity was determined by the disc diffusion method against different resistant microorganisms.

**Results:**

EAF effectively inhibited AAPH-induced oxidative damage to DNA because it can inhibit the transformation of the supercoiled form of plasmid DNA to open circular and further linear form. The oxidative hemolysis caused by AAPH in human erythrocytes was inhibited by EAF extract in a time-dependent manner, and the production of malondialdehyde (MDA) was significantly reduced, which indicates the prevention of lipid peroxidation. In antitumor assay, 76% growth of inhibition of EAC was observed compared with the control mice (*p* < 0.05) at a dose of 100 mg/kg body weight. Antimicrobial activity was evaluated against two pathogenic resistant microorganisms (*Escherichia coli* and *Pseudomonas aeruginosa*), and the highest antimicrobial activity was observed against *Pseudomonas* spp.

**Conclusion:**

EAF may have great importance in preventing oxidative damage to DNA, erythrocytes, and other cellular components as well as can be a good candidate in cancer chemotherapy and treating infectious diseases caused by antibiotic-resistant bacteria.

## 1. Introduction

Aromatic and medicinal plants are sources of diverse nutrient and non-nutrient molecules, many of which display antioxidant, anticancer, and antimicrobial properties that may protect the body against both cellular oxidation reactions and pathogens. The assembly of oxidants may be a typical event related to aerobic metabolism. When oxygen is supplied in excess or its reduction is insufficient, reactive oxygen species (ROS) or free radicals such as superoxide anions, hydroxyl radicals, and peroxide are generated [1]. Accumulation of the free radicals in body organs or tissues can cause oxidative damage to biomolecules such as DNA and erythrocytes cell, eventually resulting in many chronic diseases such as inflammatory, cancer, diabetes, aging, cardiac dysfunction, and other degenerative diseases [2].

Erythrocytes are highly sensitive to oxidative damage when exposed to reactive oxygen species (ROS) and hence are used as a cellular model to study about biomembrane integrity in relation to oxidative damage [3]. ROS induces DNA damage, as the response to free radicals with DNA involves strand break, base modification, and cross-linking of DNA-proteins. On the other hand, DNA damage caused by oxidative stress to the cell has been reported to be the main factor to develop degenerative, inflammatory diseases and accelerate cellular aging or eventually can cause cancer [4, 5].

In recent times, consumption of plant-derived phytonutrients present in berry crops, herbs, beans, oil seeds, teas, fruits, and vegetables has markedly increased [6, 7]. Since medicinal plants are being explored for different therapeutic agents for thousands of years and still are a major source for new drugs, searching potential compounds from the plants or identifying the phytochemical rich food supplements from the nature is a worthy approach to combat oxidative stress and diseases caused by such stress [8].

Antimicrobial resistance is becoming a potential threat for human beings because the number of pathogens that are resistant to commercial antibiotics is increasing day by day. On the other hand, there are few hopes and scientists are struggling for the discovery of new antibiotics. Because different classes of compounds/extracts of plants have antimicrobial activities and because 60% of the antimicrobial drugs discovered in the past few decades are of natural origin, scientists are attracted to do more research on plants for antimicrobial agents [9].

*L. rubra* comes from the family of Vitaceae and is also known as red tree shrub; it is widely found in Australia, the Malaysia and Thailand rainforest, Bangladesh, China, and India's tropical and subtropical forests at low and medium altitude [10]. Previous investigation on this plant has shown several therapeutic activities including antimicrobial and anti-inflammatory activities [11], but the available literature did not show any antitumor or protective effect against oxidative damage on the biomolecules by the plant extract. Hence, the rationale of the present study was to assess the ethyl acetate fraction (EAF) of the methanolic extract of *L. rubra* leaves for its antitumor activity and to verify the ability to inhibit the oxidative damage to human erythrocytes and DNA and antibacterial activity against resistant microorganisms.

## 2. Materials and Methods

### 2.1. Chemicals and Reagents

2′-Azobis(2-methylpropionamidine)dihydrochloride (AAPH), agarose, ethidium bromide, bovine serum albumin, gallic acid, catechin, homovanillic acid, epicatechin, chlorogenic acid, rutin hydrate, and quercetin-3-rhamnoside were purchased from Sigma-Aldrich, USA. pUC19 plasmid DNA was purchased from Genetix, Bangalore, India. The other chemicals utilized in the tests were of analytical grade and purchased from the Sigma-Aldrich and Roche.

### 2.2. Preparation of Plant Material, Extraction, and Fractionation

*L. rubra* leaves were collected from Bandarban, Bangladesh, in the month of September, 2016. The plant was authenticated by a taxonomist of the Botany Department, University of Rajshahi, and was preserved in the Phytochemistry Lab, Department of Pharmacy, University of Rajshahi, Bangladesh. After washing thoroughly with distilled water, the leaves were dried, powdered, and extracted by shaking with methanol and fractionated sequentially with *n*-hexane and ethyl acetate. Since the *n*-hexane fraction contains chlorophyll, it was not considered for further experiment. Ethyl acetate extract (EAF) was filtered through filter paper and dried under vacuum on a rotary evaporator into a thick residue. Then, it was stored in a cool and dry place for further studies.

### 2.3. Collection of Human Erythrocytes

Human erythrocytes were collected from five healthy volunteers of 18 to 40 years of age who had not taken any drug for 7 days prior to the sample collection. Blood was collected at the Medical Center of University of Rajshahi by an expert physician, preserved with anticoagulant, and centrifuged, and then erythrocytes were collected by discarding the supernatant. Collected erythrocytes were diluted in PBS.

### 2.4. Assay of the Protective Role against Oxidative Damage

Reactive oxygen species (ROS) or any oxidizing agent can damage the cellular components and cause chronic diseases. So, the capability of the extract (EAF) to neutralize or scavenge such ROS or oxidizing agents to protect DNA and erythrocytes as well as prevent lipid peroxidation was evaluated by the methods described below.

#### 2.4.1. Inhibition of AAPH-Induced Hemolysis of Human Erythrocytes

The protective activity of EAF against AAPH-induced hemolysis of human erythrocytes was determined by the method described by Yang et al. [12], with some modifications. In this test, 50 *μ*l of 5% erythrocyte suspension was mixed well with different concentrations of EAF and standard ascorbic acid and incubated for 30 min at 37°C. Then, 2, 2′-azobis-2-amidinopropane (AAPH) at a concentration of 50 mM was added to the above mixture. The reaction mixture was kept at 37°C for 6 h with frequent stirring for time-dependent hemolysis. The negative control, consisting of erythrocytes in a hypotonic buffer (100% hemolysis), was kept under the same conditions as samples. The samples were centrifuged at 1500 rpm for 10 minutes at every hour. Then, an aliquot of each supernatant was taken and diluted in PBS for taking absorbance at 540 nm. The extent of hemolysis (%) was calculated as the ratio of the absorbance at 523 nm of the sample to that of the complete hemolysis sample.

#### 2.4.2. Inhibition of Oxidative Damage on Plasmid DNA

Transformation of the supercoiled form of plasmid DNA to open circular and further linear form has been used as an index of DNA damage. The DNA damage protective activity of the EAF was assessed using pUC19 plasmid DNA [13]. 1 *μ*g of pUC19 plasmid DNA was mixed with two different concentrations of EAF (20 and 30 *μ*g/ml) and 2 *μ*l of 25 mM AAPH in PBS (pH 7.4). Then, the mixture was incubated for 30 min at 37°C and electrophoresed on 2% agarose gel containing 0.5 *μ*g/ml ethidium bromide. The band intensities were analyzed under trans-illuminating UV light, and the photo was taken using a gel documentation system.

#### 2.4.3. Inhibition of Lipid Peroxidation

Oxidation of the unsaturated lipid by ROS or any oxidizing agent can generate malondialdehyde (MDA). So, the capability of the extract to prevent such oxidation was evaluated by thiobarbituric acid reactive substances (TBARS) assay to calculate the level of MDA according to the method described by Padmaja et al. [14]. A human erythrocyte suspension, preincubated with samples at different concentrations, was used. The standard solution consisted of 500 *μ*l of 20 mM MDA in 1 ml of TBA. The absorbance was taken at 532 nm using a spectrophotometer, and the MDA level was determined using the Beer–Lambert law at a molar extinction coefficient of 156 mM−1·cm−1 and was expressed as pmol/g Hb.

### 2.5. Antitumor Activity Assay

#### 2.5.1. Experimental Tumor Model

The Department of Biochemistry, University of Rajshahi, provided transplantable tumors (Ehrlich's ascites carcinoma cells) used in this research. They were maintained in our laboratory by intraperitoneal transplantation into Swiss albino mice.

#### 2.5.2. Determination of Median Lethal Dose (LD_50_)

Various doses of EAF solution (25, 50, 100, 200, and 400 mg/kg) were given intraperitoneally in mice to determine the median lethal dose (LD_50_). The mortality was recorded after 24 h of experimental period, and for this antitumor study, 100 mg/kg dose was selected by the fixed dose method [15].

#### 2.5.3. EAC Cell Growth Inhibition Assay

To assess the cell growth inhibition properties of EAF, Swiss albino mice were divided into four groups (n = 5) and Ehrlich's ascites carcinoma (EAC) cells (1 × 106 cells/mouse) were inoculated into all mice except those in the normal group intraperitoneally on day 0. Treatments were started after 24 h of tumor inoculation and continued for 7 days. Group I served as normal and Group II as control, mice in both groups received normal saline; Group III mice received EAF at the dose of 100 mg/kg; and Group IV mice were given bleomycin (standard drug) at the dose of 3 mg/kg. The mice were sacrificed on the seventh day, and the EAC cells were collected by repeatedly washing with 0.9% saline. Then, the cells from treated and untreated mice were compared for morphological studies [16].

#### 2.5.4. Morphological Appearance of EAC Cells

The EAC cells were stained with DAPI (4, 6-diamidino-2-phenylindole) to detect the morphological changes. Visual images were taken using a fluorescent microscope. Both fluorescent and optical views were observed.

#### 2.5.5. Evaluation of Hematological Parameters

To evaluate hematological parameters, mice of all groups (four groups; *n* = 5) were injected with EAC cells (0.1 ml of 1.6 × 106 cells/mice) intraperitoneally except the normal group at day 0. After 24 hours of inoculation, normal saline (5 ml/kg/mouse/day) was administered intraperitoneally to normal (Group I) and EAC control (Group II) mice, respectively, for 10 days. EAF at 100 mg/kg/mouse/day and belomycin at 3 mg/kg/mouse/day doses were administered in Group III and Group IV, respectively. On the 12^th^ day, blood was collected from the tail vein and hematological parameters (hemoglobin, RBC, and WBC) were measured for each mice of each group [17].

### 2.6. Evaluation of Antimicrobial Activity

#### 2.6.1. Collection of Drug-Resistant Bacteria

Two drug-resistant bacteria were collected from the Microbiology Department of Rajshahi Medical College and Hospital. These bacteria were resistant against different types of common antibiotics. According to the report, *Escherichia coli* was resistant to azithromycin, cefuroxime, cefalexin, and cotrimoxazole. *Pseudomonas* is sensitive to azithromycin and cephalexin and resistant to cefuroxime, cotrimoxazole, cefepine, vancomycin, and penicillin. Here, EAF was tested alone and then given in combination with some antibiotics (azithromycin, kanamycin, penicillin, amoxicillin, and cefuroxime) to know its activity against these resistant bacteria.

#### 2.6.2. Determination of Antimicrobial Activity

The agar disc diffusion method described by Bonev et al. (2008) and Razmavar et al. (2014) [18, 19] was used for the determination of the antibacterial activity of the EAF, for which bacteria were cultured in the nutrient agar medium and each microorganism (10^6^ cells/ml) was inoculated on the surface of Mueller–Hinton agar plates. Paper discs (6 mm in diameter) saturated with the extract (50 *μ*l) and standard disc of kanamycin (30 *μ*g/disc) were placed on the surface of each inoculated plate. The plates were kept at 4°C for 5 hours and then at 37°C for 24 hours, after which it was possible to observe the zone of inhibition. Antibacterial activity was evaluated by measuring the diameter (mm) of the inhibition zone around the discs. Cultured bacteria with halos equal to or greater than 7 mm were considered susceptible to the tested sample.

### 2.7. Ethical Consideration

Swiss albino male mice of 3–4 weeks of age, weighing 23–27 g, were collected from the Animal Research Branch of Jahangirnagar University, Bangladesh. All animal studies were approved by the Ethical Committee of the Institute of Biological Sciences, University of Rajshahi, in accordance with the Guide for the Care and Use of Laboratory Animals. The protocol for using human blood cells was approved by the Institutional Animal, Medical Ethics, Biosafety, and Biosecurity Committee (IAMEBBC) for Experimentations on Animal, Human, Microbes, and Living Natural Sources at the University of Rajshahi (reference number: 31/320-IAMEBBC/IBSc).

### 2.8. Statistical Analysis

The data were analyzed by one-way ANOVA (analysis of variance) followed by multiple comparisons using Dunnett's post hoc test using SPSS software of version 16. All results were represented as mean ± standard deviation (SD). Differences at *p* < 0.05 level were considered to be statistically significant.

## 3. Results

### 3.1. Protective Role against Oxidative Damage

EAF showed significant protective activity against oxidative damage induced by AAPH to human erythrocytes and DNA as well as oxidative lipid peroxidation. The result is shown below.

#### 3.1.1. AAPH-Induced Hemolysis Assay in Human Erythrocytes

**Figure 1:**
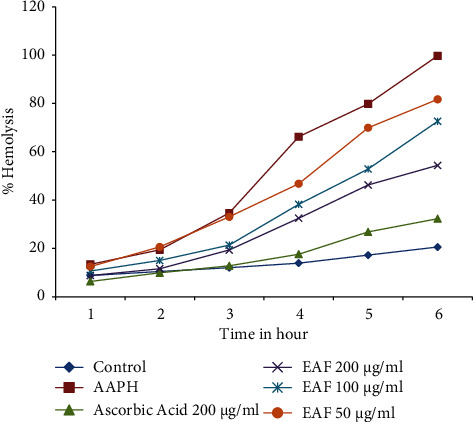
Time course effect of EAF and ascorbic acid on AAPH-induced hemolysis on erythrocytes. Values are expressed as mean ± SD of three experiments.

Figure 1 shows the protective effect of EAF against AAPH-induced hemolysis on human erythrocytes. When AAPH (50 mM) was added to the aqueous suspension of erythrocytes, time-dependent hemolysis was observed. But this hemolytic process was inhibited when the EAF (at concentrations of 50, 100, and 200 *μ*g/ml) was added in a concentration- and time-dependent manner.

#### 3.1.2. DNA Damage Protection

**Figure 2:**
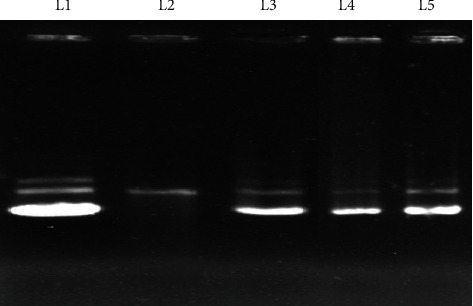
Protective effect of EAF on pUC19 plasmid DNA damage induced by AAPH. Lane 1: native DNA; Lane 2: DNA + AAPH; Lane 3: DNA + AAPH + 30 *μ*g EAF; Lane 4: DNA + AAPH + 20 *μ*g EAF; Lane 5: DNA + AAPH + 30 *μ*g gallic acid.

This assay is based on the ability of the samples to protect the DNA (P^UC19^ plasmid) against the damage caused by peroxy radicals generated by AAPH. The ability of EAF to protect oxidative damage of DNA was evaluated by analyzing the band pattern of pUC19 DNA on agarose gel as shown in Figure 2. Lane 1 shows DNA in the native supercoiled form; whereas in Lane 2, treated with AAPH, the supercoiled form has been converted into open circular DNA. Addition of EAF in Lane 3 and Lane 4 at concentrations of 30 and 20 *μ*g/ml, respectively, prevented the formation of circular form of plasmid DNA as like as the standard gallic acid (in Lane 5).

#### 3.1.3. Lipid Peroxidation Inhibition

**Figure 3:**
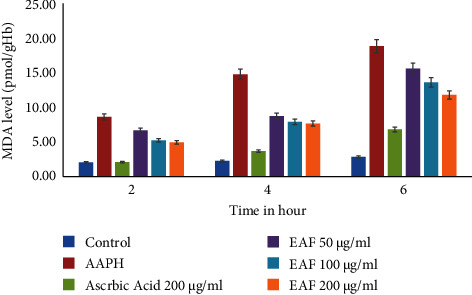
Level of MDA after treating with EAF and standard ascorbic acid. Values are expressed as mean ± SD of three experiments.

The protective activity of EAF was also evaluated by the inhibition of MDA production from the erythrocyte membrane, induced by AAPH, and the result is shown in Figure 3. MDA levels for the control-group erythrocytes were 2.06 ± 0.27, 2.27 ± 0.29, and 2.87 ± 0.15 nmol/ml, respectively, at 2, 4, and 6 h, increasing to 8.71 ± 0.34, 14.9 ± 025, and 19.02 ± 0.57 nmol/ml, respectively, after incubation with 50 mM AAPH. The addition of AAPH caused time-dependent lipid peroxidation of erythrocytes. EAF inhibited AAPH-induced MDA formation that was 4.99 ± 1.32, 7.73 ± 0.96, and 11.89 ± 1.16 nmol/ml at 2, 4, and 6 h, respectively.

### 3.2. Evaluation of Antitumor Activity

#### 3.2.1. Studies on EAC Cell Growth Inhibition

*In vivo* antitumor activity of EAF against EAC cell-bearing mice was assessed by viable EAC cells (% inhibition in cell growth). Effects of the extract on EAC cells' growth after tumor inoculation are shown in Table 1. Treatment with EAF resulted in a significant reduction of cell growth *in vivo*. The percentage of cell growth inhibition was 76.09 ± 3.21% at dose of 100 mg/kg, whereas it was 85.00 ± 5.2% with the standard anticancer drug belomycin.

#### 3.2.2. Morphological Changes of EAC Cells

**Figure 4:**
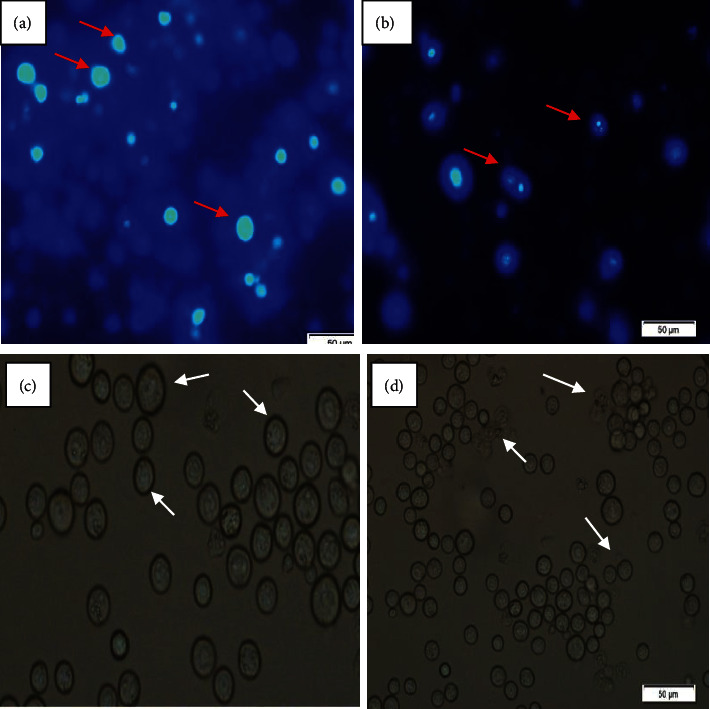
(a-b) Fluorescence and (c-d) optical microscopic observations of EAC cells for control mice and treated mice. (a, b) Fluorescence microscopic view of control and EAF-treated mice cells, (c, d) the optical microscopic view of control and EAF-treated cells, respectively. White (a) and red arrows (c) indicate normal cells. Fragmented cells (apoptotic characteristics) were indicated by red and white arrows in (b) and (d), respectively.

Changes in morphological characteristics of the EAC cells were evaluated by DAPI staining, for which the cells were collected after 7 days from both treated (with EAF 100 mg/kg/day) and nontreated EAC-bearing mice. In the control group (solvent treated), the nuclei of the EAC cells were round, regular, and homogeneously stained with DAPI as shown in Figure 4. At the same time, apoptotic morphologic alterations like membrane and nuclear condensation were also noted in EAF-treated EAC cells.

#### 3.2.3. Studies on Hematological Parameters

Hematological parameters of untreated EAC cell-bearing mice showed significant (*P* < 0.05) changes when compared with normal mice (Table 2). The total WBC count was found to increase with a reduction in the hemoglobin content and total RBC count. At the same time interval, treatment with EAF could bring back these altered parameters to almost normal values. The overall results of this study clearly demonstrated the antitumor activity of EAF against EAC.

### 3.3. Antimicrobial Activity

Evaluation of the antibacterial activity of EAF was tested against two drug-resistant bacteria *E. coli* and *P. aeruginosa* by the disc diffusion method. The results of this assay are shown in Table 3, where EAF at all three concentrations showed dose-dependent activity against both organisms and the maximum zone of inhibition was 10 mm for *E. coli* and 11 mm for *P. aeruginosa* compared with 6 mm and 12 mm, respectively, for the standard kanamycin.

Combination of EAF with the standard kanamycin also showed an increased activity compared with both EAF and standard alone, where the maximum zone of inhibition for combined therapy was 10 mm for *E. coli* and 16 mm for *P. aeruginosa* as shown in Table 3.

## 4. Discussion

The main focus of the study was to investigate the EAF of the extract of *L. rubra* leaves for its inhibition capacity against ROS to prevent the damage caused by such free radicals to cellular components such as DNA, cell membrane, and erythrocytes [20], as well as antitumor and antibacterial properties. Previous phytochemical studies with leaves of this plant showed an abundance of phenolic constituents such as flavonoids, leucoanthocyanidins, p-hydroxybenzoic acid, syringic acid, and gallic acid [21]. So, the presence of such naturally occurring compounds indicates the potential of the plant to act against oxidative stress-related damage to DNA and erythrocytes as well as the capacity to prevent tumor cell growth. The antimicrobial activity of the plant extracts is mainly exhibited by secondary metabolites such as alkaloids, tannins, terpenoids, alkaloids, and flavonoids [22, 23]. The presence of such compounds in *L. rubra* in preliminary screening also made the plant extracts a suitable candidate for antimicrobial screening.

Protection against oxidative damage to DNA and erythrocytes was evaluated by AAPH-induced hemolysis protection in human erythrocytes, inhibition of MDA production from membrane lipid peroxidation, and prevention of oxidation-induced cleavage in DNA strands. The findings of these experiments showed some promising results where EAF was found to prevent hemolysis in a dose-dependent manner. The maximum level of MDA after treating with AAPH at 6 h was 19.02 ± 0.57 nmol/ml, which was significantly reduced to 11.89 ± 1.16 nmol/ml after treating with EAF. The gel electrophoresis image also showed brighter bands of DNA with increasing concentrations of EAF. So, the above instances indicate the significant protective role of the extract and therefore can be used in preventing chronic diseases due to alteration of cellular components such as cancer, aging, and neurodegenerative diseases [3]. The findings were similar to the study performed by Reddy et al. in another species of this plant family, *Leea indica* [23].

In the next step, we investigated its role in cell growth inhibition on EAC-bearing Swiss albino mice. The EAC cells are experimental tumor models used worldwide in cancer research [10]. EAC cells collected from EAF-treated mice showed some morphological changes such as breakage of inner cell membrane, cells shrinkage, chromosomal condensation, and nuclear fragmentation when observed under a fluorescence microscope, which are also the important and reliable criteria for judging the potency of any drug as anticancer agents. These morphological changes are the hallmark of the apoptosis of EAC cells, which suppress the tumor development. In the absence of apoptosis process, abnormal cell proliferation may occur that can lead to cancer development [24]. On the other hand, EAC cells were normal in control mice. So, in our experiment, EAF extract could inhibit the cell growth along with morphological features of apoptosis. The effectiveness of the extract against EAC cell-bearing mice has further been verified by monitoring the changes in hematological and biological parameters. In our study, reduction in RBC or % hemoglobin in tumor-bearing mice may occur, which is mainly due to iron deficiency or hemolytic or myelopathic conditions [25]. The extract could significantly recover the hemoglobin content; RBC and WBC cell count indicate the protective action on the hemopoietic system. All these are measured and are very important aspects in justifying the effectiveness of EAF in cancer chemotherapy.

Antibiotic resistance has been a great issue all around the world from developing to developed world, and it is predicted that if such trend continues, treatment for infectious diseases will be more challenging [26, 27]. Several studies have demonstrated the antimicrobial activity of the species of the genus *Leea*, where Harun et al. [28] reported significant activity of *L. indica* extract to be effective against two organisms, *S. aureus* and *S. epidermidis*; Islam et al. [29] showed a considerable activity of *L. macrophylla* against Gram-negative bacteria and fungi; and Khan et al. [30] reported such activity of *L. tetramera* against fungi.

A similar result was found in our study, where the activity was assessed against two resistant organisms. An increase in the diameter of the zone of inhibition with an increase in dose indicates considerable activity of the plant extract against resistant bacteria. The effect was better when used in combination with the existent antibiotic (kanamycin) and provided a possible hope to combat antimicrobial resistance.

## 5. Conclusion

The potential of the EAF of *L. rubra* leaf extract to prevent oxidative damage of the cellular components has been found to be significant. At the same time, its effect on the inhibition of tumor cell growth was notable, and hence it can be a potential candidate for further investigation for anticancer drugs. Activity against resistant organisms was considerable, but better activity was reported while used in combination with standard antibiotics.

## Figures and Tables

**Table 1:** Effects of EAF on viable Ehrlich's ascites carcinoma (EAC) cell growth.

Treatment group	Dose (mg/kg/day)	No. of viable EAC cells (×10^7^ cells/ml)	% of cell growth inhibition
Control	—	61.14 ± 2.23	—
EAF	100	14.17 ± 1.97^*∗*^	76.09 ± 3.21^*∗*^
Bleomycin	3	13.78 ± 1.58	85.00 ± 5.2

Data are expressed as mean ± SD (*n* = 5). Analysis of variance followed by LSD and Dunnett't post hoc test (IBM-SPSS/20); ^*∗*^*P* < 0.05: significant difference with respect to EAC control.

**Table 2:** Effects of EAF and standard bleomycin on blood parameters of tumor-bearing and normal mice.

Treatment	% of Hb (g/dl)	RBC (×10^9^ cells/ml)	WBC (×10^6^ cells/ml)
Normal	13.26 ± 0.12	5.98 ± 0.27	10.05 ± 0.42
EAC + vehicle	8.41 ± 0.19^*∗*^	2.21 ± 0.168^*∗*^	120.7 ± 1.81^*∗*^
EAC + EAF (100 mg/kg)	10.47 ± 0.19^t^	3.67 ± 0.156^t^	42.38 ± 1.92^t^
Belomycin (3 mg/kg)	11.94 ± 1.05	4.01 ± 0.11^t^	35.12 ± 1.56^t^

Data are expressed as mean ± SD for five animals in each group. Analysis of variance followed by LSD and Dunnett't post hoc test (IBM-SPSS/20); ^*∗*^*P*  <  0.05: against normal group, and ^#^*P* < 0.05: against EAC control group.

**Table 3:** Diameter of zone of inhibition (mm) of EAF against drug-resistant bacteria.

	Zone of inhibition (mm)				
Bacteria	Kanamycin-30 (standard)	EAF (50 *μ*g/disc)	EAF (100 *μ*g/disc)	EAF (200 *μ*g/disc)	EAF (100 *μ*g/disc) + kanamycin-30
*E. coli*	6	5	8	10	10
*P. aeruginosa*	12	6	8	11	16

## Data Availability

The data used to support the findings of this study are available from the corresponding author upon request.
